# (1*R*,3*S*)-3-Hydroxy­meth­yl-*N*-isopropyl-2,2-dimethyl­cyclo­propane­carboxamide

**DOI:** 10.1107/S1600536809051538

**Published:** 2009-12-12

**Authors:** Jiangchun Zhong, Bing Zheng, Shicong Hou

**Affiliations:** a Department of Applied Chemistry, China Agriculture University, 100193, Beijing, People’s Republic of China

## Abstract

The asymmetric unit of the title compound, C_10_H_19_NO_2_, prepared from (−)-1*R*-*cis*-caronaldehyde, contains two independent mol­ecules. In the crystal structure, inter­molecular O—H⋯O and O—H⋯N hydrogen bonds form an extensive three-dimensional hydrogen-bonding network.

## Related literature

For details of the synthesis, see Huang *et al.* (2001 ▶). For the crystal structures of related derivatives of (−)-1*R*-*cis*-caronaldehyde, see: Na & Wang (2009 ▶); Wang *et al.* (2009 ▶).
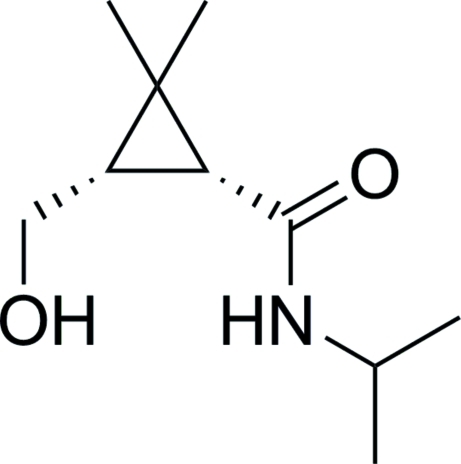


## Experimental

### 

#### Crystal data

C_10_H_19_NO_2_
*M* *_r_* = 185.26
Orthorhombic, 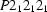

*a* = 9.4628 (19) Å
*b* = 12.298 (3) Å
*c* = 18.710 (4) Å
*V* = 2177.4 (8) Å^3^
*Z* = 8
Cu *K*α radiation
μ = 0.62 mm^−1^
*T* = 173 K
0.53 × 0.43 × 0.39 mm

#### Data collection

Rigaku R-AXIS Rapid IP area-detector diffractometer
Absorption correction: multi-scan (*ABSCOR*; Higashi, 1995 ▶) *T*_min_ = 0.734, *T*_max_ = 0.794
15043 measured reflections
2271 independent reflections
2129 reflections with *I* > 2σ(*I*)
*R*_int_ = 0.046

#### Refinement

*R*[*F* ^2^ > 2σ(*F* ^2^)] = 0.042
*wR*(*F* ^2^) = 0.110
*S* = 1.06
2271 reflections
240 parameters
H-atom parameters constrained
Δρ_max_ = 0.26 e Å^−3^
Δρ_min_ = −0.19 e Å^−3^

### 

Data collection: *RAPID-AUTO* (Rigaku, 2001 ▶); cell refinement: *RAPID-AUTO*; data reduction: *RAPID-AUTO*; program(s) used to solve structure: *SHELXS97* (Sheldrick, 2008 ▶); program(s) used to refine structure: *SHELXL97* (Sheldrick, 2008 ▶); molecular graphics: *XP* in *SHELXTL* (Sheldrick, 2008 ▶); software used to prepare material for publication: *SHELXL97*.

## Supplementary Material

Crystal structure: contains datablocks I, New_Global_Publ_Block. DOI: 10.1107/S1600536809051538/cv2626sup1.cif

Structure factors: contains datablocks I. DOI: 10.1107/S1600536809051538/cv2626Isup2.hkl

Additional supplementary materials:  crystallographic information; 3D view; checkCIF report

## Figures and Tables

**Table 1:** Hydrogen-bond geometry (Å, °)

*D*—H⋯*A*	*D*—H	H⋯*A*	*D*⋯*A*	*D*—H⋯*A*
O1—H1*A*⋯O3^i^	0.84	1.96	2.796 (3)	174
O3—H3⋯O1^ii^	0.84	1.98	2.782 (3)	160
N1—H1⋯O4	0.88	2.03	2.906 (3)	176
N2—H2⋯O2^iii^	0.88	2.09	2.964 (3)	171

Symmetry codes: (i) 
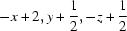
; (ii) 
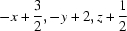
; (iii) 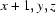.

**Fig. 1.**
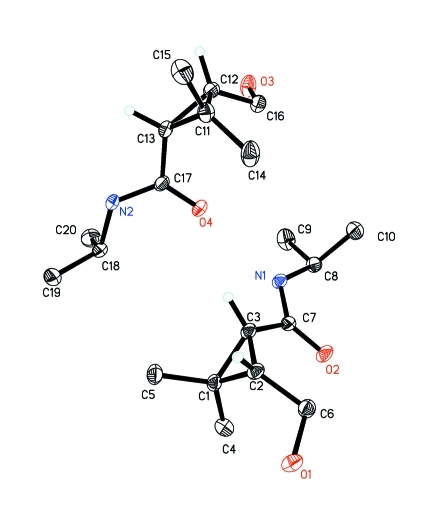
Two independent molecules of (I), with atomic labels and 30% probability displacement ellipsoids. Some of H atoms omitted for clarity.

## supplementary crystallographic information

### Comment

In continuation of our study of new (-)-1*R*-*cis*-caronaldehyde derivatives (Na & Wang, 2009; Wang *et al.*, 2009), we present here the crystal structure of the title compound (I).

The asymmetric unit of (I) contains two independent molecules (Fig. 1). The crystal structure is stabilized by intermolecular O—H···O and O—H···N hydrogen bonds (Table 1).

### Experimental

We have synthesized the title compound following the known procedure (Huang *et al.*, 2001).

(1*R*,5*S*)-4-hydroxy-6,6-dimethyl-3-oxa-bicyclo[3.1.0]-hex-an-2-one (2.84 g, 20 mmol) was dissolved in the mixture of 30 ml of Et_2_O and 5 ml of MeOH. This solution was cooled to 0°C and a solution of diazomethane in ether was added. The solution was slowly allowed to warm to room temperature without additional heating. The reaction mixture was concentrated under reduced pressure. The crude product was passed through a silica gel column (hexane/ethyl acetate = 6:1) to afford (1*R*,3*S*)-Methyl-3-formyl-2,2-dimethylcyclopropane-carboxylate as a colorless oil (2.9 g, 93% yield). [α]^D^_18_= -73.4(*c* 0.99, CHCl_3_); IR (neat) 2957, 1731, 1701, 1439, 1379, 1200, 1137, 1119, 839 cm^-1^; ^1^H NMR (500 MHz, CDCl_3_): *δ* 1.27 (s,3*H*), 1.55 (s, 3H), 1.83–1.86 (m, 1H), 2.12 (d, 1H, *J* = 9.5 Hz), 3.71 (s, 3H),9.75 (d, 1H, *J* = 9.5 Hz,); ^13^CNMR (125 MHz, CDCl_3_): *δ* 15.5, 28.3, 29.8, 36.1, 40.9, 52.2, 170.4,200.4; HRMS (TOF): m/*z* calcd for C_8_H_13_NO_3_ [*M*^+^H^+^]: 157.0859, found: 157.0865.

NaBH_4_ (3.78 g, 100 mmol) was dissolved in 60 ml of dry MeOH,and a solution of (1*R*,3*S*)-Methyl-3-formyl-2,2-dimethylcyclopropane-carboxylate (15.6 g,100 mmol) in MeOH was added slowly into above mixture. After it, the reaction was kept for 30 min. Then, saturated HCl (1 ml) was added to stop the reactionand extracted with ether (40 ml x3). The organic layer was dried over anhydrousNa_2_SO_4_. Then, concentrated to give alight yellow oil (13.74 g, 87% yield). To a solution of benzene (60 ml) was added the light yellow oil (8 g, 50 mmol), the mixture was refluxed for 2 h. Then, concentrated under reduced pressure (2 m mH g, 56°C) to give (1*R*,5*S*)-6,6-Dimethyl-3-oxa-bicyclo[3.1.0]hexan-2-one with 90% yield. ^1^HNMR (500 MHz, DMSO): *δ* 1.176 (3*H*, s),1.184 (3*H*, s), 1.943–1.958 (1*H*,m), 2.034–2.059 (1*H*, m), 4.14–4.16 (d, 1H, *J* = 9.5 Hz), 4.350–4.381(1*H*, m); ^13^C NMR (125 MHz, DMSO): *δ* 14.37, 22.99, 25.18, 30.01, 30.47, 66.49, 174.92.

The isopropanamine (2.2 ml, 20 mmol) was dissolved in dry THF (10 ml), cooled to -15°C, and, DIBAL-H (13 ml, 20 mmol) was injected. After themixture was sirred for 20 min, the solution was allowed to warm to 30°C and to react for 3 h. Then, it was cooled to -5 °C, and a solution of (1*R*,5*S*)-6,6-Dimethyl-3-oxa-bicyclo[3.1.0]hexan-2-one (2.2 ml) in THF (7.5 ml) was added withstirring for 10 min. After 20 h at room temperture, the reaction was quenched with water (10 ml) and 4 *N* HCl (15 ml) and the mixture was extracted several times with Et_2_O. The combined organic phases were washed with 1 N HCl, then, dried over Na_2_SO_4_ and concentrated under reduced pressure to give the products (1*R*,3*S*)-*N*-isopropyl-3-(hydroxymethyl)-2,2-\ dimethylcyclopropanecarboxamide (3.48 g, 87% yield). [α]^D^_20_= 41.0 (*c* 1.14, CHCl_3_); IR(KBr): 3272.57, 2960.39, 1654.66, 1630.67, 1547.28, 1450.94, 1240.11, 1198.22,1120.70, 1027.72, 699.99 cm^-1.1^H NMR (500 MHz, DMSO): *δ* 1.144–1.200 (3*H*, s), 1.372–1.428 (3*H*, s), 1.657 (2*H*,m), 3.123–3.150 (1*H*,m), 3.840–3.883 (1*H*, m), 3.972–4.028 (1*H*, m), 4.442–4.458 (2*H*, m), 6.067 (1*H*, m), 7.264–7.302 (3*H*, m), 7.333–7.363 (2*H*, m). ^13^CNMR (125 MHz, DMSO): *δ* 15.48,24.15, 28.59, 31.81, 32.59, 43.80, 59.08, 127.56, 127.76, 128.74,138.18, 171.26.

### Refinement

All H atoms were positioned geometrically and treated as riding on their parent atoms, with C—H = 0.98 - 1.00 Å, O—H = 0.84 Å, and with *U*_iso_ (H) = 1.2-1.5*U*_eq_ of the parent atom. In the absence of any significant anomalous scatterers in the molecule, the 1671 Friedel pairs were merged before the final refinement.

### Crystal data

**Table:**

C_10_H_19_NO_2_	*D*_x_ = 1.130 Mg m^−^^3^
*M**_r_* = 185.26	Cu *K*α radiation, λ = 1.54186 Å
Orthorhombic, *P*2_1_2_1_2_1_	Cell parameters from 426 reflections
*a* = 9.4628 (19) Å	θ = 2.2–68.3°
*b* = 12.298 (3) Å	µ = 0.62 mm^−^^1^
*c* = 18.710 (4) Å	*T* = 173 K
*V* = 2177.4 (8) Å^3^	Block, colourless
*Z* = 8	0.53 × 0.43 × 0.39 mm
*F*(000) = 816	

### Data collection

**Table:**

Rigaku R-AXIS Rapid IP area-detector diffractometer	2271 independent reflections
Radiation source: rotating anode	2129 reflections with *I* > 2σ(*I*)
graphite	*R*_int_ = 0.046
ω scans at fixed χ = 45°	θ_max_ = 68.2°, θ_min_ = 4.3°
Absorption correction: multi-scan (*ABSCOR*; Higashi, 1995)	*h* = −11→1
*T*_min_ = 0.734, *T*_max_ = 0.794	*k* = −14→14
15043 measured reflections	*l* = −22→22

### Refinement

**Table:**

Refinement on *F*^2^	Hydrogen site location: inferred from neighbouring sites
Least-squares matrix: full	H-atom parameters constrained
*R*[*F*^2^ > 2σ(*F*^2^)] = 0.042	*w* = 1/[σ^2^(*F*_o_^2^) + (0.0502*P*)^2^ + 1.0161*P*] where *P* = (*F*_o_^2^ + 2*F*_c_^2^)/3
*wR*(*F*^2^) = 0.110	(Δ/σ)_max_ < 0.001
*S* = 1.06	Δρ_max_ = 0.26 e Å^−^^3^
2271 reflections	Δρ_min_ = −0.19 e Å^−^^3^
240 parameters	Extinction correction: *SHELXL*, Fc^*^=kFc[1+0.001xFc^2^λ^3^/sin(2θ)]^-1/4^
Primary atom site location: structure-invariant direct methods	Extinction coefficient: 0.0058 (5)
Secondary atom site location: difference Fourier map	

### Special details

**Table:**

Geometry. All esds (except the esd in the dihedral angle between two l.s. planes) are estimated using the full covariance matrix. The cell esds are taken into account individually in the estimation of esds in distances, angles and torsion angles; correlations between esds in cell parameters are only used when they are defined by crystal symmetry. An approximate (isotropic) treatment of cell esds is used for estimating esds involving l.s. planes.
Refinement. Refinement of F^2^ against ALL reflections. The weighted R-factor wR and goodness of fit S are based on F^2^, conventional R-factors R are based on F, with F set to zero for negative F^2^. The threshold expression of F^2^ > 2sigma(F^2^) is used only for calculating R-factors(gt) etc. and is not relevant to the choice of reflections for refinement. R-factors based on F^2^ are statistically about twice as large as those based on F, and R- factors based on ALL data will be even larger.

### Fractional atomic coordinates and isotropic or equivalent isotropic displacement parameters (Å^2^)

**Table:**

	*x*	*y*	*z*	*U*_iso_*/*U*_eq_	
O1	0.6210 (2)	1.20449 (16)	0.05202 (10)	0.0338 (5)	
H1A	0.6936	1.2425	0.0454	0.041*	
O2	0.5838 (2)	0.94520 (18)	0.20498 (10)	0.0365 (5)	
O3	1.1281 (2)	0.81763 (16)	0.47435 (11)	0.0408 (5)	
H3	1.0658	0.8016	0.5045	0.049*	
O4	1.0762 (2)	0.8858 (2)	0.28306 (11)	0.0440 (6)	
N1	0.7727 (2)	0.87180 (18)	0.26112 (11)	0.0273 (5)	
H1	0.8639	0.8795	0.2689	0.033*	
N2	1.2844 (2)	0.88054 (19)	0.22414 (11)	0.0286 (5)	
H2	1.3750	0.8966	0.2234	0.034*	
C1	0.8241 (3)	1.0016 (2)	0.08897 (14)	0.0287 (6)	
C2	0.7827 (3)	1.1031 (2)	0.12682 (14)	0.0281 (6)	
H2A	0.8595	1.1589	0.1275	0.034*	
C3	0.8184 (3)	1.0024 (2)	0.17075 (13)	0.0280 (6)	
H3A	0.9138	1.0058	0.1936	0.034*	
C4	0.7151 (4)	0.9388 (2)	0.04695 (15)	0.0370 (7)	
H4A	0.6261	0.9369	0.0737	0.055*	
H4B	0.7487	0.8644	0.0391	0.055*	
H4C	0.6996	0.9744	0.0007	0.055*	
C5	0.9697 (3)	0.9970 (3)	0.05666 (17)	0.0428 (8)	
H5A	0.9664	1.0248	0.0076	0.064*	
H5B	1.0030	0.9215	0.0562	0.064*	
H5C	1.0345	1.0416	0.0851	0.064*	
C6	0.6372 (3)	1.1523 (2)	0.12027 (14)	0.0320 (6)	
H6A	0.5649	1.0947	0.1256	0.038*	
H6B	0.6228	1.2062	0.1589	0.038*	
C7	0.7133 (3)	0.9388 (2)	0.21314 (13)	0.0264 (6)	
C8	0.6914 (3)	0.8114 (2)	0.31424 (14)	0.0297 (6)	
H8A	0.5976	0.7929	0.2929	0.036*	
C9	0.7659 (4)	0.7061 (3)	0.33356 (18)	0.0479 (8)	
H9A	0.7639	0.6567	0.2925	0.072*	
H9B	0.7176	0.6720	0.3741	0.072*	
H9C	0.8641	0.7216	0.3466	0.072*	
C10	0.6663 (3)	0.8800 (3)	0.38007 (15)	0.0389 (7)	
H10A	0.6260	0.9502	0.3660	0.058*	
H10B	0.7561	0.8920	0.4049	0.058*	
H10C	0.6004	0.8423	0.4120	0.058*	
C11	1.2353 (3)	1.0709 (2)	0.37272 (15)	0.0321 (6)	
C12	1.2361 (3)	0.9684 (2)	0.41652 (14)	0.0282 (6)	
H12A	1.3165	0.9641	0.4511	0.034*	
C13	1.2821 (3)	0.9638 (2)	0.33829 (13)	0.0279 (6)	
H13A	1.3869	0.9586	0.3326	0.033*	
C14	1.0943 (4)	1.1208 (3)	0.35373 (18)	0.0494 (9)	
H14A	1.0811	1.1881	0.3809	0.074*	
H14B	1.0184	1.0696	0.3655	0.074*	
H14C	1.0918	1.1370	0.3025	0.074*	
C15	1.3528 (4)	1.1522 (3)	0.38644 (18)	0.0465 (8)	
H15A	1.3133	1.2176	0.4086	0.070*	
H15B	1.3979	1.1716	0.3411	0.070*	
H15C	1.4231	1.1198	0.4185	0.070*	
C16	1.1006 (3)	0.9219 (2)	0.44476 (15)	0.0307 (6)	
H16A	1.0611	0.9705	0.4819	0.037*	
H16B	1.0307	0.9156	0.4056	0.037*	
C17	1.2042 (3)	0.9076 (2)	0.28063 (14)	0.0289 (6)	
C18	1.2337 (3)	0.8174 (2)	0.16345 (14)	0.0315 (6)	
H18A	1.1303	0.8316	0.1576	0.038*	
C19	1.3086 (3)	0.8541 (3)	0.09619 (15)	0.0442 (8)	
H19A	1.2926	0.9321	0.0891	0.066*	
H19B	1.2714	0.8138	0.0551	0.066*	
H19C	1.4101	0.8403	0.1008	0.066*	
C20	1.2542 (4)	0.6969 (2)	0.17724 (17)	0.0427 (8)	
H20A	1.2034	0.6761	0.2208	0.064*	
H20B	1.3551	0.6814	0.1832	0.064*	
H20C	1.2174	0.6552	0.1366	0.064*	

### Atomic displacement parameters (Å^2^)

**Table:**

	*U*^11^	*U*^22^	*U*^33^	*U*^12^	*U*^13^	*U*^23^
O1	0.0275 (10)	0.0340 (10)	0.0398 (10)	−0.0030 (9)	−0.0071 (9)	0.0073 (9)
O2	0.0173 (10)	0.0533 (13)	0.0389 (10)	−0.0032 (9)	0.0001 (8)	0.0135 (10)
O3	0.0351 (12)	0.0369 (11)	0.0504 (12)	0.0051 (10)	0.0140 (10)	0.0123 (10)
O4	0.0170 (9)	0.0769 (16)	0.0381 (11)	−0.0034 (11)	−0.0003 (8)	−0.0046 (11)
N1	0.0200 (11)	0.0331 (12)	0.0289 (11)	−0.0030 (10)	−0.0005 (9)	0.0055 (10)
N2	0.0175 (10)	0.0384 (12)	0.0299 (11)	−0.0025 (10)	0.0008 (9)	−0.0020 (10)
C1	0.0235 (14)	0.0336 (14)	0.0290 (13)	0.0024 (12)	0.0038 (11)	0.0071 (12)
C2	0.0211 (13)	0.0326 (14)	0.0306 (13)	−0.0036 (12)	−0.0021 (11)	0.0038 (12)
C3	0.0183 (13)	0.0355 (15)	0.0303 (13)	−0.0028 (11)	0.0002 (10)	0.0056 (12)
C4	0.0430 (17)	0.0373 (15)	0.0306 (13)	−0.0019 (14)	0.0012 (13)	0.0005 (12)
C5	0.0321 (17)	0.0566 (19)	0.0397 (16)	0.0110 (15)	0.0106 (13)	0.0122 (16)
C6	0.0296 (14)	0.0322 (14)	0.0342 (14)	0.0035 (12)	0.0012 (12)	0.0016 (12)
C7	0.0222 (13)	0.0326 (13)	0.0245 (12)	−0.0019 (11)	−0.0005 (11)	−0.0001 (11)
C8	0.0271 (15)	0.0330 (15)	0.0291 (13)	−0.0041 (12)	0.0012 (11)	0.0057 (11)
C9	0.059 (2)	0.0370 (16)	0.0481 (18)	0.0053 (17)	0.0123 (17)	0.0107 (14)
C10	0.0395 (17)	0.0424 (16)	0.0348 (15)	0.0007 (15)	0.0027 (13)	0.0011 (13)
C11	0.0330 (15)	0.0273 (13)	0.0359 (14)	0.0038 (12)	0.0047 (13)	0.0027 (11)
C12	0.0248 (14)	0.0315 (14)	0.0284 (12)	0.0008 (12)	−0.0006 (11)	0.0024 (11)
C13	0.0196 (13)	0.0321 (14)	0.0319 (13)	0.0012 (12)	0.0022 (11)	−0.0002 (11)
C14	0.048 (2)	0.0483 (18)	0.0523 (19)	0.0195 (18)	0.0151 (16)	0.0168 (16)
C15	0.056 (2)	0.0368 (16)	0.0469 (18)	−0.0090 (16)	0.0116 (17)	−0.0012 (14)
C16	0.0282 (15)	0.0316 (14)	0.0322 (13)	0.0024 (12)	0.0026 (12)	0.0042 (12)
C17	0.0180 (13)	0.0384 (15)	0.0304 (13)	0.0011 (11)	−0.0017 (11)	0.0059 (12)
C18	0.0208 (13)	0.0446 (16)	0.0291 (13)	−0.0036 (13)	−0.0025 (11)	0.0002 (12)
C19	0.0355 (17)	0.067 (2)	0.0299 (14)	−0.0078 (16)	0.0013 (13)	0.0012 (15)
C20	0.0419 (19)	0.0409 (16)	0.0454 (17)	−0.0066 (15)	−0.0034 (15)	−0.0043 (14)

### Geometric parameters (Å, °)

**Table:**

O1—C6	1.437 (3)	C9—H9A	0.9800
O1—H1A	0.8400	C9—H9B	0.9800
O2—C7	1.237 (3)	C9—H9C	0.9800
O3—C16	1.421 (3)	C10—H10A	0.9800
O3—H3	0.8400	C10—H10B	0.9800
O4—C17	1.242 (3)	C10—H10C	0.9800
N1—C7	1.342 (3)	C11—C12	1.504 (4)
N1—C8	1.460 (3)	C11—C14	1.511 (4)
N1—H1	0.8800	C11—C15	1.517 (4)
N2—C17	1.343 (3)	C11—C13	1.531 (4)
N2—C18	1.457 (3)	C12—C16	1.500 (4)
N2—H2	0.8800	C12—C13	1.528 (4)
C1—C2	1.488 (4)	C12—H12A	1.0000
C1—C5	1.506 (4)	C13—C17	1.478 (4)
C1—C4	1.510 (4)	C13—H13A	1.0000
C1—C3	1.531 (4)	C14—H14A	0.9800
C2—C6	1.509 (4)	C14—H14B	0.9800
C2—C3	1.525 (4)	C14—H14C	0.9800
C2—H2A	1.0000	C15—H15A	0.9800
C3—C7	1.493 (4)	C15—H15B	0.9800
C3—H3A	1.0000	C15—H15C	0.9800
C4—H4A	0.9800	C16—H16A	0.9900
C4—H4B	0.9800	C16—H16B	0.9900
C4—H4C	0.9800	C18—C19	1.513 (4)
C5—H5A	0.9800	C18—C20	1.516 (4)
C5—H5B	0.9800	C18—H18A	1.0000
C5—H5C	0.9800	C19—H19A	0.9800
C6—H6A	0.9900	C19—H19B	0.9800
C6—H6B	0.9900	C19—H19C	0.9800
C8—C10	1.512 (4)	C20—H20A	0.9800
C8—C9	1.518 (4)	C20—H20B	0.9800
C8—H8A	1.0000	C20—H20C	0.9800
			
C6—O1—H1A	106.9	H10A—C10—H10B	109.5
C16—O3—H3	110.2	C8—C10—H10C	109.5
C7—N1—C8	123.2 (2)	H10A—C10—H10C	109.5
C7—N1—H1	117.1	H10B—C10—H10C	109.5
C8—N1—H1	117.3	C12—C11—C14	118.2 (2)
C17—N2—C18	124.0 (2)	C12—C11—C15	117.1 (3)
C17—N2—H2	120.5	C14—C11—C15	114.8 (3)
C18—N2—H2	115.3	C12—C11—C13	60.44 (17)
C2—C1—C5	117.7 (3)	C14—C11—C13	120.3 (3)
C2—C1—C4	119.8 (2)	C15—C11—C13	115.2 (2)
C5—C1—C4	113.4 (3)	C16—C12—C11	120.5 (2)
C2—C1—C3	60.66 (17)	C16—C12—C13	124.5 (2)
C5—C1—C3	115.7 (2)	C11—C12—C13	60.68 (17)
C4—C1—C3	120.0 (2)	C16—C12—H12A	113.7
C1—C2—C6	122.6 (2)	C11—C12—H12A	113.7
C1—C2—C3	61.09 (17)	C13—C12—H12A	113.7
C6—C2—C3	124.9 (2)	C17—C13—C12	125.1 (2)
C1—C2—H2A	112.9	C17—C13—C11	124.4 (2)
C6—C2—H2A	112.9	C12—C13—C11	58.88 (17)
C3—C2—H2A	112.9	C17—C13—H13A	112.8
C7—C3—C2	124.3 (2)	C12—C13—H13A	112.8
C7—C3—C1	123.5 (2)	C11—C13—H13A	112.8
C2—C3—C1	58.25 (17)	C11—C14—H14A	109.5
C7—C3—H3A	113.3	C11—C14—H14B	109.5
C2—C3—H3A	113.3	H14A—C14—H14B	109.5
C1—C3—H3A	113.3	C11—C14—H14C	109.5
C1—C4—H4A	109.5	H14A—C14—H14C	109.5
C1—C4—H4B	109.5	H14B—C14—H14C	109.5
H4A—C4—H4B	109.5	C11—C15—H15A	109.5
C1—C4—H4C	109.5	C11—C15—H15B	109.5
H4A—C4—H4C	109.5	H15A—C15—H15B	109.5
H4B—C4—H4C	109.5	C11—C15—H15C	109.5
C1—C5—H5A	109.5	H15A—C15—H15C	109.5
C1—C5—H5B	109.5	H15B—C15—H15C	109.5
H5A—C5—H5B	109.5	O3—C16—C12	109.0 (2)
C1—C5—H5C	109.5	O3—C16—H16A	109.9
H5A—C5—H5C	109.5	C12—C16—H16A	109.9
H5B—C5—H5C	109.5	O3—C16—H16B	109.9
O1—C6—C2	110.4 (2)	C12—C16—H16B	109.9
O1—C6—H6A	109.6	H16A—C16—H16B	108.3
C2—C6—H6A	109.6	O4—C17—N2	121.8 (3)
O1—C6—H6B	109.6	O4—C17—C13	124.1 (3)
C2—C6—H6B	109.6	N2—C17—C13	114.1 (2)
H6A—C6—H6B	108.1	N2—C18—C19	109.6 (2)
O2—C7—N1	122.4 (2)	N2—C18—C20	110.3 (2)
O2—C7—C3	124.1 (2)	C19—C18—C20	111.9 (3)
N1—C7—C3	113.4 (2)	N2—C18—H18A	108.3
N1—C8—C10	110.7 (2)	C19—C18—H18A	108.3
N1—C8—C9	110.6 (2)	C20—C18—H18A	108.3
C10—C8—C9	110.8 (2)	C18—C19—H19A	109.5
N1—C8—H8A	108.2	C18—C19—H19B	109.5
C10—C8—H8A	108.2	H19A—C19—H19B	109.5
C9—C8—H8A	108.2	C18—C19—H19C	109.5
C8—C9—H9A	109.5	H19A—C19—H19C	109.5
C8—C9—H9B	109.5	H19B—C19—H19C	109.5
H9A—C9—H9B	109.5	C18—C20—H20A	109.5
C8—C9—H9C	109.5	C18—C20—H20B	109.5
H9A—C9—H9C	109.5	H20A—C20—H20B	109.5
H9B—C9—H9C	109.5	C18—C20—H20C	109.5
C8—C10—H10A	109.5	H20A—C20—H20C	109.5
C8—C10—H10B	109.5	H20B—C20—H20C	109.5
			
C5—C1—C2—C6	139.5 (3)	C14—C11—C12—C16	−4.3 (4)
C4—C1—C2—C6	−5.2 (4)	C15—C11—C12—C16	139.9 (3)
C3—C1—C2—C6	−115.0 (3)	C13—C11—C12—C16	−115.0 (3)
C5—C1—C2—C3	−105.5 (3)	C14—C11—C12—C13	110.8 (3)
C4—C1—C2—C3	109.8 (3)	C15—C11—C12—C13	−105.0 (3)
C1—C2—C3—C7	−111.2 (3)	C16—C12—C13—C17	−3.9 (4)
C6—C2—C3—C7	0.1 (4)	C11—C12—C13—C17	−112.5 (3)
C6—C2—C3—C1	111.4 (3)	C16—C12—C13—C11	108.6 (3)
C2—C1—C3—C7	112.7 (3)	C12—C11—C13—C17	113.6 (3)
C5—C1—C3—C7	−138.6 (3)	C14—C11—C13—C17	6.2 (4)
C4—C1—C3—C7	3.2 (4)	C15—C11—C13—C17	−138.2 (3)
C5—C1—C3—C2	108.7 (3)	C14—C11—C13—C12	−107.3 (3)
C4—C1—C3—C2	−109.5 (3)	C15—C11—C13—C12	108.2 (3)
C1—C2—C6—O1	−74.7 (3)	C11—C12—C16—O3	171.1 (2)
C3—C2—C6—O1	−150.0 (2)	C13—C12—C16—O3	97.7 (3)
C8—N1—C7—O2	−8.4 (4)	C18—N2—C17—O4	−4.6 (4)
C8—N1—C7—C3	172.5 (2)	C18—N2—C17—C13	175.1 (2)
C2—C3—C7—O2	16.9 (4)	C12—C13—C17—O4	22.4 (4)
C1—C3—C7—O2	−54.9 (4)	C11—C13—C17—O4	−51.1 (4)
C2—C3—C7—N1	−164.0 (2)	C12—C13—C17—N2	−157.3 (2)
C1—C3—C7—N1	124.3 (3)	C11—C13—C17—N2	129.2 (3)
C7—N1—C8—C10	−85.8 (3)	C17—N2—C18—C19	147.4 (3)
C7—N1—C8—C9	151.0 (3)	C17—N2—C18—C20	−89.0 (3)

### Hydrogen-bond geometry (Å, °)

**Table:**

*D*—H···*A*	*D*—H	H···*A*	*D*···*A*	*D*—H···*A*
O1—H1A···O3^i^	0.84	1.96	2.796 (3)	174
O3—H3···O1^ii^	0.84	1.98	2.782 (3)	160
N1—H1···O4	0.88	2.03	2.906 (3)	176
N2—H2···O2^iii^	0.88	2.09	2.964 (3)	171

Symmetry codes: (i) −*x*+2, *y*+1/2, −*z*+1/2; (ii) −*x*+3/2, −*y*+2, *z*+1/2; (iii) *x*+1, *y*, *z*.
